# Introduction of the Modified Neuroanatomy Motivation Questionnaire and Its Role in Comparing Medical Student Attitudes Towards Learning Neuroanatomy Between Neuro-enthusiasts and Standard Students

**DOI:** 10.1007/s40670-021-01371-2

**Published:** 2021-10-14

**Authors:** Samuel Hall, Octavia Kurn, Deepika Anbu, Eva Nagy, Oliver Dean, Alistair Robson, Charles Taylor, Ahmad Elmansouri, Kate Geoghegan, December Payne, Matthew Myers, Jonny Stephens, Wassim Merzougui, Scott Border

**Affiliations:** Centre for Learning Anatomical Sciences, Faculty of Medicine, University of Southampton, Southampton General Hospital, Tremona Road, Southampton, SO16 6YD UK

**Keywords:** Neuroanatomy, Undergraduate medical education, Neurophobia, Student motivation

## Abstract

**Background:**

Neurophobia has been identified as a potential barrier to adequate knowledge of neurology in the medical community, and therefore to patient safety. There is a drive to identify the source of neurophobia, in the hope of tackling it. Comparing the learning motivations of standard medical students with those who enjoy neuroanatomy may be a way of doing this.

**Methods:**

The science motivation questionnaire (SMQ) was modified for neuroanatomy. It was distributed to three cohorts of second year medical students and students attending the extracurricular National Undergraduate Neuroanatomy Competition (NUNC). Cohen’s *D* test for effect size was used to compare standard medical students and those attending the NUNC.

**Results:**

Five hundred ninety-seven questionnaires were completed by second year students, and 320 by NUNC attendees. The differences in motivation to learn neuroanatomy between the 2 groups mainly fell into themes of career motivation, personal relevance, intrinsic motivation and assessment anxiety.

**Conclusion:**

This study has demonstrated the use of the SMQ in neuroanatomy, and found differences in motivators to learn neuroanatomy between self-selecting “neurophiles” and standard medical students, mainly relating to intrinsic motivation and its role in their lives. More research is needed to further explore these differences and how they might apply to interventions in medical school curricula.

## Introduction

Neurophobia is a term used to describe the fear that medical students have of neurology-based subjects [1]. Neurological conditions are common; thus, neurophobia has the potential to negatively impact upon the safety of patients in the UK and the numbers of doctors entering neurology or neurosurgery.

It has been suggested that neurophobia begins at medical school, and may even be made worse as a result of engagement with neuroscience modules [2, 3]. The explanations for why neurophobia develops include insufficient time given to neurology within medical school curricula, and basic neuroscience teaching not integrated with clinical understanding or skills [4]. Learning the subject of neuroanatomy in particular has been identified as the most common sub-discipline associated with the cause of neurophobia amongst UK medical students [5]. Whilst a lack of neuroanatomy knowledge and neurophobia are not synonymous, it is likely that one is a factor which causes the other. The aversion to studying neuroanatomy is most likely due to its intricate details and complex 3D relationships [6, 7]. Many measures such as computer-based learning, student conferences [8] and extended courses [4] aimed to stimulate interest in the subject have been proposed as methods to potentially tackle the fear of learning neuroanatomy.

The National Undergraduate Neuroanatomy Competition (NUNC) is an annual event in the UK whereby medical students volunteer to sit neuroanatomy exams in their free time. The format of the NUNC has been previously described [9, 10]. The success of this competition demonstrates that a self-selecting cohort of medical students who have a passion for neuroanatomy currently exists, and are therefore presumably unaffected by neurophobia. Identifying differences in motivating factors towards studying neuroanatomy between NUNC students and “standard” medical students may allow educators to further understand how neurophobia manifests amongst “standard” student populations and facilitate suitable practices to promote pro-neuroanatomy motivating factors. The aim of this study was to compare the differences in learning motivations towards neuroanatomy between “standard” and neurophilic medical students.

## Methods

### Survey Instrument

The Science Motivation Questionnaire (SMQ) is a validated survey developed by Glynn et al. [11]. The SMQ contains 30 questions with 5 underlying factors. A second version (SMG II) [12] has also been produced; however, this has a reduced number of questions relating to examination anxiety. Given that examinations are a key source of anxiety amongst medical students [13], the first-generation SMQ was selected for this study.

The SMQ was modified by the first author so that its wording was relevant to neuroanatomy and medical careers rather than general science. The modified Neuroanatomy Motivation Questionnaire (NMQ) was then internally reviewed by the remaining authors for content validity using a Delphi technique. The final modified version is shown in the Appendix.

### Survey Distribution

The modified NMQ was distributed as a paper-based questionnaire to 3 consecutive cohorts of second year (BM5 BMBS) medical students (*n* = 257, 263 and 267) at the University of Southampton. The questionnaires were distributed at the end of the neuroanatomy/head and neck module following their final examination. The same questionnaire was also distributed to all the attendees for 3 consecutive NUNCs. These questionnaires were completed by both groups for the academic years 2017/18, 2018/19, and 2019/20. The responses of University of Southampton medical students who also competed in NUNC were kept in both cohorts to avoid causing selection bias.

Internal validity was confirmed using Cronbach alpha and item-total score correlations. The modified questionnaire scored 0.83 on the CBA test which is acceptable for basic research tools but not so high (i.e. > 0.9) to suggest redundancy within the questionnaire [14]. The item-total correlations are all > 0.3 with the exception of question 30 (Table 1).

**Table 1:** The item-total score correlations and extracted factors

Original factor	Survey item	Item-total correlation	Extracted factors							
			1	2	3	4	5	6	7	8
Intrinsic motivation and personal relevance	I find learning neuroanatomy interesting	0.53				0.812				
	I enjoy learning neuroanatomy	0.56				0.787				
	The neuroanatomy I learn has practical usefulness to me	0.50							0.801	
	The neuroanatomy I learn has relevance to me	0.46							0.868	
	The neuroanatomy I learn is more important to me than grades	0.41		0.31						
	The neuroanatomy I learn relates to my personal goals	0.56		0.476						
	I like neuroanatomy that challenges me	0.62				0.574				
	Understanding neuroanatomy gives me a sense of accomplishment	0.39				0.545				
	I think about how I will use neuroanatomy I learn	0.56		0.693						
	I think about how the neuroanatomy I learn will be helpful to me as a doctor	0.51		0.718						
Self-efficacy and assessment anxiety	I am nervous about how I will do on neuroanatomy tests	0.32	0.854							
	I worry about failing neuroanatomy tests	0.40	0.883							
	I become anxious when it is time to take a neuroanatomy test	0.36	0.849							
	I am confident I will do well on neuroanatomy tests	0.51					0.583			
	I am concerned that other students are better at neuroanatomy	0.30	0.595							
	I believe I can earn a top grade in the neuroanatomy course	0.56					0.644			
	I hate taking neuroanatomy tests	0.37	0.560							
	I believe I can master the knowledge in the neuroanatomy course	0.57					0.648			
	I am confident I will do well in the neuroanatomy dissection lab	0.57					0.689			
Self-determination	I put effort into learning neuroanatomy	0.41			0.862					
	I prepare well for neuroanatomy tests	0.42			0.877					
	I use strategies that ensure I learn neuroanatomy well	0.50			0.791					
	If I am having trouble learning neuroanatomy, I try to figure out why	0.47			0.522					
Career motivation	I think about how learning neuroanatomy can help my career	0.55		0.843						
	I think about how neuroanatomy can help me get a good job	0.51		0.793						
Grade motivation	I like to do better than the other students on neuroanatomy	0.41						0.766		
	Earning a good neuroanatomy grade is important to me	0.39						0.694		
	I expect to do as well as or better than other students in the neuroanatomy course	0.47						0.560		
	I think about how my neuroanatomy grade will affect my overall year grade	0.31								0.660
	It is my fault if I do not understand neuroanatomy	0.11								0.782

Principal component analysis was performed to demonstrate consistency of underlying factors compared to the original SMQ using the second year medical students as the baseline cohort. The suitability of the correlation matrix was confirmed with a Bartlett’s test of sphericity = 7125.4, df 435, *p* < 0.0001 and a KMO measure of sampling adequacy = 0.856. The PCA extracted 8 factors with an eigenvalue > 1 (Table 1) which explained 65.2% of the total variance.

Comparing the standard students with NUNC students was performed using Cohen’s *D* to calculate effect sizes. All statistical tests were performed using SPSS version 26 (IBM, New York, USA), except for Cohen’s *D* which was performed using an online calculator [15].

## Results

### Baseline Performance of the Modified Neuroanatomy Motivation Questionnaire

The three cohorts of second year medical students returned 597 completed questionnaires. The distribution of responses over the 3 years was as follows: 2017/18 *n* = 214, 2018/19 *n* = 164 and 2019/20 *n* = 219 with response rates of 83.3%, 62.4% and 82.0% respectively. The student’s total scores on the questionnaire were normally distributed with mean = 98.4 (SD = 13.7) which is comparable to that described for the Glynn Science Motivation Questionnaire [11].

Visual inspection demonstrates that the 8 factors in the current extraction are directly comparable to the 5 factors found in the original Glynn Science Motivation Questionnaire (intrinsic motivation, career motivation, self-determination, self-efficacy and grade motivation). The additional 3 factors are divisions of previous factors. Factor 2 which focuses on *career motivation* aligns with items on *personal relevance* from Glynn’s first factor. This interpretation demonstrates that the underlying themes measured by the original questionnaire are also present in the modified questionnaire for neuroanatomy.

### Differences in Motivations Between Regular Medical Students and NUNC Attendees

Over the 3 years for which the NMQ was distributed to the NUNC attendees (2018 *n* = 126, 2019 *n* = 127, 2020 *n* = 118), 338 questionnaires were returned (response rate 91.2%). Fifteen students attended NUNC more than 1 year in which case their first questionnaire was used for analysis and the repeat attempts (*n* = 18) were removed. Three hundred and twenty questionnaires were included in the final analysis.

The total scores on the modified Glynn questionnaire were significantly higher for those students attending NUNC than the baseline cohort of 2nd year medical students (111.1 ± 13.0 v 98.4 ± 13.7, *p* < 0.0001). NUNC students thus had a different motivation profile than general medical students at Southampton University.

There was a gender difference in the total modified Glynn questionnaire scores. Both the male and female subgroups scored higher on the questionnaire than their respective genders in the standard cohort (113.6 v 103.6, *p* < 0.001 and 106.2 v 99.3, *p* < 0.01). Male students attending NUNC scored significantly higher than female NUNC students (113.6 v 106.2, *p* = 0.03). There was no significant difference in the male and female overall scores in the standard group.

In order to determine which of the 30 elements in the questionnaire were responsible for the difference in overall motivation between the two groups, Cohen’s *D* for effect size was calculated for each element. Fifteen of the 30 questionnaire elements showed a Cohen’s *D* score greater than 0.5 between the NUNC and general medical students (Table 2). Career motivation, personal relevance, intrinsic motivation and assessment anxiety were themes showing the biggest difference between the two groups. Self-determination and competition against other students did not show noteworthy differences between standard and NUNC students.

**Table 2:** Cohen’s *D* for effect size comparing NUNC students against the baseline cohort of second year medical students (Cohen’s *D* > 0.5 in italics)

Original factor	Questionnaire element	Cohen’s *D*	
Intrinsic motivation and personal relevance	*I find learning neuroanatomy interesting*	*0.754*	*Medium*
	*I enjoy learning neuroanatomy*	*0.951*	*Large*
	The neuroanatomy I learn has practical usefulness to me	0.004	Very small
	The neuroanatomy I learn has relevance to me	0.138	Very small
	*The neuroanatomy I learn is more important to me than grades*	*0.665*	*Medium*
	*The neuroanatomy I learn relates to my personal goals*	*0.889*	*Large*
	*I like neuroanatomy that challenges me*	*0.939*	*Large*
	Understanding neuroanatomy gives me a sense of accomplishment	0.459	Small
	*I think about how I will use the neuroanatomy I learn*	*0.529*	*Medium*
	*I think about how the neuroanatomy I learn will be helpful to me as a doctor*	*0.508*	*Medium*
Self-efficacy and assessment anxiety	*I am nervous about how I will do on neuroanatomy tests*	*0.65*	*Medium*
	*I worry about failing neuroanatomy tests*	*0.841*	*Large*
	*I become anxious when it is time to take a neuroanatomy test*	*0.676*	*Medium*
	I am confident I will do well on neuroanatomy tests	0.433	Small
	I am concerned that other students are better at neuroanatomy	0.121	Very small
	I believe I can earn a top grade in the neuroanatomy course	0.491	Small
	*I hate taking neuroanatomy tests*	*0.788*	*Medium*
	I believe I can master the knowledge in the neuroanatomy course	0.286	Small
	I am confident I will do well in the neuroanatomy dissection lab	0.393	Small
Self-determination	I put effort into learning neuroanatomy	0.103	Very small
	I prepare well for neuroanatomy tests	0.087	Very small
	I use strategies that ensure I learn neuroanatomy well	0.009	Very small
	If I am having trouble learning neuroanatomy, I try to figure out why	0.114	Very small
Career motivation	*I think about how learning neuroanatomy can help my career*	*0.739*	*Medium*
	*I think about how neuroanatomy can help me get a good job*	*0.748*	*Medium*
Grade motivation	*I like to do better than the other students on neuroanatomy*	*0.523*	*Medium*
	Earning a good neuroanatomy grade is important to me	0.166	Very small
	I expect to do as well as or better than other students in the neuroanatomy course	0.403	Small
	I think about how my neuroanatomy grade will affect my overall year grade	0.364	Small
	*It is my fault if I do not understand neuroanatomy*	*0.819*	*Large*

## Discussion

It has been over 2 decades since the term neurophobia was first coined to describe the phenomenon of medical students’ aversion to neurological disorders [1]. The root causes for neurophobia have been speculated to be difficulty with learning neuroanatomy, poor teaching approaches and issues with neuroscience education [5, 16]. Previous educational initiatives to combat the phenomenon include hosting undergraduate conferences [8] and extending the length of neurology modules [4].

The current research into neurophobia has focused on identifying the negative elements associated with neuroanatomy teaching and learning of the subject. However, little attention has been paid to the medical students who feel positively towards neuroanatomy and enjoy it. This group of “neurophiles” may possess traits or mindsets which could provide insight into how and why neurophobia occurs in a standard student. Previous work has shown that doctors with positive university experiences of neuroscience are more likely to consider working in “brain-related specialties” [3]. Thus, change in educational practices may be able to enhance these neurophilic motivators.

The SMQ adapted for this study was initially designed to measure the separate facets underpinning students’ motivation towards studying science subjects. It was modified for applicability towards neuroanatomy motivation as a tool to probe the underpinning attitudes of medical students towards neuroanatomy. The modified item phrasing was first assessed on a cohort of 3 years’ worth of second year medical students following their neuroanatomy module. The response rates for each cohort were high showing no selection bias in the students used for validating the NMQ. The Cronbach alpha of 0.83 confirms a high degree of internal validity and supports the use of the modified neuroanatomy questionnaire in medical students.

The PCA broadly followed the same pattern as the results found in the original work by Glynn et al. [11]. However, this study extracted 8 factors compared to the original work which only identified 5. *Intrinsic motivation and personal relevance* were initially combined but separated out in our analysis, with two question items from this section better aligning with the *career motivation* theme. This difference may be because the original questionnaire referred to science in general, whereas neuroanatomy, as with all medical knowledge, has a direct link to clinical ability and thus career performance in medicine. *Self-efficacy and exam anxiety* were split cleanly into the two separate components in our analysis. *Grade motivation* was also split into two factors, with a new factor of competition against fellow medical students appearing alongside *grade motivation*. This is perhaps unsurprising, given the competitive nature of medical degrees compared with some other science-based subjects. Whilst there are some subtle differences in how the factors extract, the underlying themes are the same between the Glynn Science Motivation Questionnaire and the current modified questionnaire for neuroanatomy motivation.

The NUNC is a unique medical student competition which, by its voluntary nature, self-selects students who are interested in neuroanatomy and thus can serve as a cohort of neurophiles to use as subjects for this study. The new NMQ was applied to the students at the NUNC as an exploratory analysis to identify which motivators were different amongst the NUNC students compared to standard medical students.

The NUNC students demonstrated a Cohen’s *D* score of > 0.5 for the questionnaire items associated with *personal relevance*, *intrinsic motivation*, *exam anxiety* and *career motivation*. NUNC is attended by many medical students who wish to pursue careers in neurology and neurosurgery; thus, it is natural that studying neuroanatomy has a greater *personal relevance* for the NUNC cohort. The difference in *intrinsic motivation* is likely to be due to an individual’s personal interests and indirect relationships to personal relevance and career motivation, in that those wishing to pursue a career in the area will likely find the topic more interesting than those who do not. All of these factors are incorporated in the umbrella of autonomous/internal motivation (as opposed to controlled/external motivation) within self-determination theory [17]. Intrinsic motivation, the prototypical subtype of autonomous motivation [18], has a significant influence on learning engagement and academic performance [19], and thus is a key target when looking to modify student attitudes.

Achievement goal theory and performance orientation describe how outperforming peers motivates learning [20] and a study on medical, dentistry and chiropractic students identified 80% of students wanted to outperform their peers on their anatomy examinations [21]. It may be expected that NUNC students with aspirations towards careers that are perceived as competitive and intelligent [22] are more goal orientated than standard medical students. However, in this study, this was not the case and the *grade motivation* theme was not different between the cohorts. Grade motivation and performance orientation is thus a consistent personality trait across medical students and not influenced by the students’ feelings towards neuroanatomy. Grade motivation would be a type of external regulation motivation within self-determination theory [18], and the lack of difference seen in this study compounds the idea that the differences between neurophiles and standard students lie in the intrinsic motivators. However, within *grade motivation*, there was an interesting large difference for the item *It is my fault if I don’t understand neuroanatomy* which perhaps suggests a more personally responsible attitude to learning the subject given their desire to pursue neuroscience careers.

There is mixed evidence on whether self-efficacy amongst heath science students correlates with examination performance [23, 24]. Interestingly, *self-efficacy* and *self-determination* were not different between the neurophile and standard cohorts in this study. This is surprising given that neurophobia is felt to be due, in part, to the difficulty of learning neuroanatomy [16]. If neurophiles and standard students have the same self-belief in their ability to master neuroanatomy, then neurophobia is more than just a fear over having to master a difficult subject. It is possible that neurophiles also perceive neuroanatomy as a difficult subject but are motivated to find methods to overcome these difficulties in order to master the subject.

Taken together, the results of this study suggest that NUNC students are different than standard medical students in how they perceive the role of neuroanatomy in their external world and the impact this has on autonomous motivation, whereas the students’ internal relationship between their mindset (as measured by self-efficacy and self-determination) with the learning of neuroanatomy is not different between the two groups. It can therefore be speculated that educational reforms which target this external relationship in order to maximise intrinsic motivation could be the most fruitful approaches in averting neurophobia. A programme encouraging more responsibility for learning, perhaps with more self-directed study time and highlighting personal relevance, may also aid this approach. The importance of self-determination theory’s autonomous motivation in student learning has already been recognised by other educationalists [25].

The factors causing neurophobia have been described as modifiable and non-modifiable [26]. The former relates to how neuroanatomy is taught and the students’ perceptions; the latter relates to previous experiences and preconceptions. Educational reforms targeting modifiable factors such as placing neuroanatomy in clinical, real-world situations may increase the students’ feeling of *personal relevance* [27] which could be coupled with near-peer teaching to maximise learner-friendly terminology through cognitive congruence [28]. Computer games which increase personal relevance by putting students in real-world emergency medicine situations increase student motivation and time spent studying [29] which is an approach which could also be applied to neuroanatomy. Non-modifiable factors such as preconceptions could still be modified by intervening earlier in the student’s career and introducing the student to the role of neuroanatomy in their future medical lives, before the negative preconceptions are formed. This may be able to be achieved, for example, through summer schools for secondary school students interested in a career in medicine in the future.

One limitation of this study is the generalisability of the NMQ. The baseline cohort of standard medical students was selected from a single university, and so generalisability could be increased by including more universities. Secondly, since neurophobia is a concept and there is no measure or test to confirm it, we have no way of confirming that neurophobia exists in the standard university students against whom NUNC participants were compared. Given that the purpose of this study was exploratory analysis, both of these limitations can be rectified using larger, more diverse, samples. Further to this, the data was all self-reported rather than directly collected by a researcher which would improve response rate and data accuracy. All of the questionnaires were distributed following examinations, and perceived performance on these examinations may influence students’ feeling towards their neuroanatomy motivation. Lastly, the responses from 2nd year students in the University of Southampton cohort were anonymous and it is not possible to remove duplicate responses from students repeating the year.

## Conclusions

This study is the first to explore the modified Neuroanatomy Motivation Questionnaire for neuroanatomy in medical students. Furthermore, the exploratory analysis demonstrates that there are differences in underlying motivations towards studying neuroanatomy between NUNC students and a standard cohort. These initial differences are in how the students view the role of neuroanatomy in their lives. The NUNC provides a unique cohort of medical students for investigating neurophobia, and further research can develop these observed differences.

## Appendix

**Table 3:** The final modified version of the NMQ.

	Always	Never	Rarely	Sometimes	Usually
I find learning neuroanatomy interesting					
I enjoy learning neuroanatomy					
The neuroanatomy I learn has practical usefulness for me as a medical student					
The neuroanatomy I learn is relevant to me as a medical student					
The neuroanatomy I learn is more important to me than the grade I receive					
The neuroanatomy I learn relates to my personal goals in medicine					
I like neuroanatomy that challenges me					
Understanding neuroanatomy gives me a sense of accomplishment					
I think about how I will use the neuroanatomy I learn					
I think about how the neuroanatomy I learn will be helpful to me as a doctor					
I am nervous about how I will do on neuroanatomy tests					
I worry about failing neuroanatomy tests					
I become anxious when it is time to take a neuroanatomy test					
I am confident I will do well on neuroanatomy tests					
I am concerned that other students are better at neuroanatomy					
I believe I can earn a top grade in the neuroanatomy course					
I hate taking neuroanatomy tests					
I believe I can master the knowledge in the neuroanatomy course					
I am confident I will do well in the neuroanatomy dissection lab					
I put enough effort into learning neuroanatomy					
I prepare well for neuroanatomy tests					
I use strategies that ensure I learn neuroanatomy well					
If I am having trouble learning neuroanatomy, I try to figure out why					
I think about how learning neuroanatomy can help my career					
I think about how learning neuroanatomy can help me get a good job					
I like to do better than the other students on neuroanatomy tests					
Earning a good neuroanatomy grade is important to me					
I expect to do as well as or better than other students in my neuroanatomy course					
I think about how my neuroanatomy grade will affect my overall year grade					
It is my fault if I do not understand neuroanatomy					
